# The Effects of Doxapram Blocking the Response of Gram-Negative Bacterial Toxin (LPS) at Glutamatergic Synapses

**DOI:** 10.3390/biology12081046

**Published:** 2023-07-25

**Authors:** Kaitlyn E. Brock, Robin L. Cooper

**Affiliations:** Department of Biology, University of Kentucky, Lexington, KY 40506-0225, USA; kaitlynbrock@uky.edu

**Keywords:** glutamatergic receptor, immune, lipopolysaccharides, neuromuscular junction, neuron, synapse

## Abstract

**Simple Summary:**

The actions of bacterial toxin lipopolysaccharides (LPS) can lead to the development of illness in humans and animals by triggering an immune system response. LPS is known to negatively affect ion channels within mammalian cells and block receptors in flies. There are currently no pharmacological blockers for LPS. In recent studies, the compound doxapram has been shown to block some of the negative effects of LPS. In the crayfish model, LPS is thought to increase transmission at neuromuscular junctions. The effects of doxapram on the crayfish model are relatively unknown. This study aimed to determine the general effect of doxapram on the crayfish model and if doxapram could also block the ability of LPS to increase transmission at synapses. It was shown that high concentrations of doxapram rapidly decreased transmission, while lower concentrations increased transmission for a short time and then decreased transmission. When exposed to a combination of LPS and doxapram, the increase in transmission typically seen with LPS did not occur and transmission was completely decreased. These results could suggest that LPS and doxapram are working through the same pathways. This study provides further information regarding bacterial infections and how pharmacological agents may affect their development.

**Abstract:**

Lipopolysaccharides (LPS) associated with Gram-negative bacteria are one factor responsible for triggering the mammalian immune response. Blocking the action of LPS is key to reducing its downstream effects. However, the direct action of LPS on cells is not yet fully addressed. LPS can have rapid, direct effects on cells in the absence of a systemic immune response. Recent studies have shown that doxapram, a blocker of a subset of K2P channels, also blocks the acute actions of LPS. Doxapram was evaluated to determine if such action also occurs at glutamatergic synapses in which it is known that LPS can increase synaptic transmission. Doxapram at 5 mM first enhanced synaptic transmission, then reduced synaptic response, while 10 mM rapidly blocked transmission. Doxapram at 5 mM blocked the excitatory response induced by LPS. Enhancing synaptic transmission with LPS and then applying LPS combined with doxapram also resulted in retarding the response of LPS. It is possible doxapram and LPS are mediated via a similar receptor or cellular responses. The potential of designing pharmacological compounds with a similar structure to doxapram and determining the binding of such compounds can aid in addressing the acute, direct actions by LPS on cells.

## 1. Introduction

The rapid and direct actions of bacterial toxins are complicated to address due to several constituents of bacteria, which can each have their own effect and induce immune reactions, such as a heightened release of inflammatory cytokines, within an organism. Thus, secondary effects can mask or add to the initial rapid effects of bacterial toxins. Clinical treatments of animals as well as humans who have septicemia due to Gram-negative bacteria can be complicated due to the combinatorial effects. The focus in clinical treatment remains on addressing the secondary immune responses. Reducing the action of induced secondary responses is key; however, there are no known blockers to many of the receptors of the bacterial toxins. Though the receptor complex for lipopolysaccharides (LPS) is known to involve a Toll-like receptor 4 (TLR4) and form a receptor complex referred to as CD14/TLR4/MD2, ref. [1] there are no known pharmacological blockers. Invertebrate models, such as the fruit fly (*Drosophila melanagaster*) and horseshoe crab, have served a vital role in addressing the immune response induced by LPS in mammals and ultimately led to a Nobel Prize (i.e., Hoffmann and Beutler). It is advantageous to continue pursuing comparative physiological studies in other animals (i.e., insects and crustaceans) to address actions induced by bacterial toxins and to screen for pharmacological interventions [2,3,4,5].

In insects, Gram-negative bacteria are known to have genomic actions through the peptidoglycan recognition proteins (PGRP-LE/PGRP-LC receptors) and the immune deficiency (IMD) signaling pathway [6,7,8,9]. The IMD cascade is linked to the NF-κB factor Relish by the genomic response to produce several antimicrobial peptides (AMPs) in the hemolymph of insects and crustaceans (see review [9]). As for mammals, there are no known pharmacological blockers of the PGRP-LE/PGRP-LC receptors to reduce the downstream effects of LPS response. In crustaceans, these receptors and cascades have yet to be fully investigated; thus, the cellular responses and mechanisms of bacterial toxins such as LPS still need to be addressed [8].

One of the major constituents of the Gram-negative bacterial toxins known to initiate an immune response is LPS. Not all the cellular effects of LPS appear to be mediated via the receptor complex mentioned above. There are also direct actions on ion channels such as reducing potassium currents in mammalian cells [10] and blocking glutamatergic receptors to reduce synaptic efficacy in *Drosophila* [11]. In addition, it is of interest to address the rapid acute responses to long-term LPS exposure which likely recruits secondary immune reactions. The innate immune system in insects and other invertebrates promotes a rapid aggregation and clotting of hemolymph when exposed to Gram-negative bacteria or isolated LPS [12]. The hemolymph isolated from horseshoe crabs was a gold standard, known as the *Limulus* amebocyte lysate assay, as it is sensitive and promotes a reaction when detecting the presence of Gram-negative bacteria. This assay was used for screening the cleanliness of surgical tools and prosthetics to be implanted in humans [13,14,15]. If LPS, as a compound, can avoid being caught by the innate immunity response in the hemolymph of invertebrates and reach tissues, there appears to also be a rapid action of LPS on tissues. This has been demonstrated by using isolated nerves and muscles as well as cardiac tissues flushed with saline to remove any hemolymph after the tissue has been removed from the animal or held in situ [11,16,17].

The direct action of LPS on the larval *Drosophila* heart and neuromuscular junctions is speculated to alter the membrane potential by transiently activating a K^+^ channel subtype known as a K2P (two-P-domain K^+^ subunits) subtype, which helps to maintain the resting membrane potential of the cells [2,18]. The compound, doxapram (trade names, Stimulex or Respiram), blocked the acute actions of LPS from *Serratia marcescens* [11,19]. Recently, it was demonstrated that LPS from *Serratia marcescens* also has rapid effects on the NMJs of crayfish but with different effects than those reported for the NMJs of the larval *Drosophila*. Thus, the focus of this investigation was to determine the response of the NMJ in the crayfish model (i.e., the opener muscle in the walking legs) to exposure of doxapram and to determine if doxapram would block the responses normally induced by LPS. The LPS from *Serratia marcescens* was used to directly compare to previous reports on crayfish NMJs and to the effects on the larval *Drosophila* and amphibian NMJs as well as hippocampal slices of rodents [16]. In addition, *Serratia marcescens* is a bacterial strain known to cause septicemia in invertebrates and humans [20,21,22,23] and is common in the same aquatic environments as crayfish [24,25].

## 2. Materials and Methods

### 2.1. Animals

Experiments were performed using Red Swamp Crayfish (*Procambarus clarkii*). They were obtained from a distribution center in Atlanta, GA, USA, then delivered to and bought from a local supermarket in Lexington, KY, USA. Some were bought directly from Kyle LeBlanc Crawfish Farms, 302 Saint Peter St., Raceland, LA USA, 70394. Throughout the study, mid-sized crayfish measuring 6–10 cm in body length and 12.5–25 g in body weight were used. Each animal was housed in individual standardized plastic containers with weekly exchanged dry fish food and aerated water (20–21 °C).

### 2.2. Neuromuscular Junction

**Figure 1:**
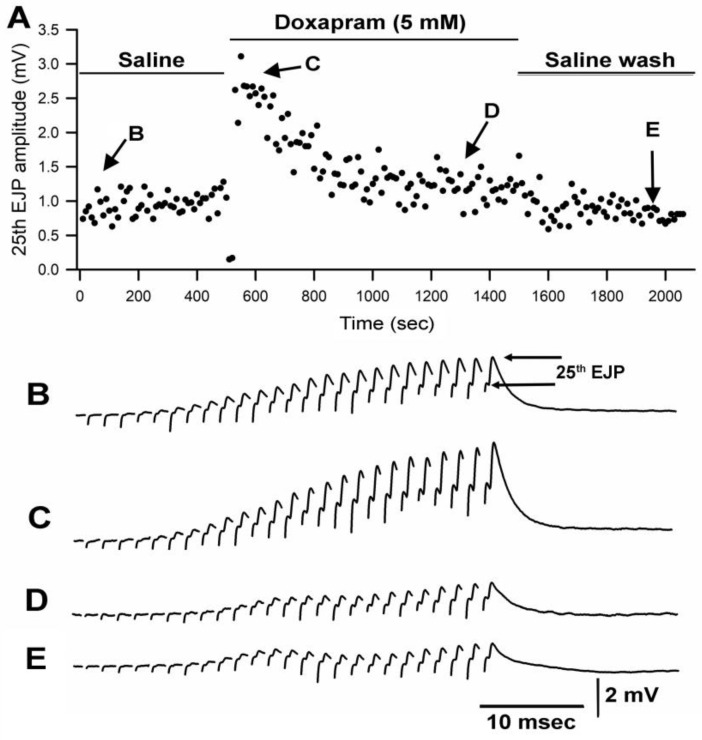
Doxapram at 5 mM transiently enhances synaptic transmission followed by depression. (**A**) is the amplitude of the 25th EJP in a 40 Hz stimulus train over time before, during, and after exposure to doxapram. (**B**–**E**) are representative responses obtained at the times indicated in (**A**). The amplitude of the 25th EJPs are measured from the procedure trough to the peak response as shown in (**B**).

Details of the dissection and electrophysiological recordings of the opener neuromuscular junction of the walking legs are described in video format [26]. The short-term facilitation was induced by providing a train of 25 or 40 stimuli at 40 Hz or 60 Hz, respectively. The excitatory nerve was stimulated with a suction electrode in an isolated motor nerve in the meropodite region of the leg. Intracellular excitatory junction potential (EJPs) recordings were performed by standard procedures [27]. Analysis of responses used the amplitudes of the EJPs from the short-term facilitation train of pulses from the 25 or 40 stimuli at 40 Hz or 60 Hz, respectively, as previously described [27]. The recording details and general experimental procedures examining the effects of LPS have been described previously [11,16,17]. The amplitude of the 25th EJPs are measured from the proceeding trough to the peak response (Figure 1B).

### 2.3. Chemicals

Commercial LPS from *Serratia marcescens* was dissolved in physiological saline the day of experimentation. This LPS may also contain some associated peptidoglycans from *Serratia marcescens*. The crayfish saline used is a modified Van Harreveld’s solution (in mM: 205 NaCl, 5.3 KCl, 13.5 CaCl_2_·2H_2_O, 2.45 MgCl_2_·6H_2_O and 5 HEPES adjusted to pH 7.4). A 500 μg/mL measure of LPS was used in all experiments. Doxapram and LPS were dissolved directly in saline to be used. All chemicals listed above were obtained from Sigma-Aldrich (St. Louis, MO, USA).

### 2.4. Statistical Analysis

This analysis was performed with SigmaStat software. *p* of ≤0.05 is considered statistically significant. Normality Test (Shapiro–Wilk) and Equal Variance Test (Brown–Forsythe) were performed by the software. All pairwise multiple comparison procedures used the post analysis with a Bonferroni *t*-test. Paired *t*-test and Sign test were also used for statistical analysis. Averaged data are expressed as a mean (±SEM).

## 3. Results

### 3.1. Effect of Doxapram Enhancing and Depressing Synaptic Transmission

Since the crayfish neuromuscular junction (NMJ) had not been investigated previously for the effect of doxapram, two different concentrations of doxapram were used to examine the outcome. In a previous report, doxapram at 10 mM was needed to block the action of LPS at the larval *Drosophila* NMJ [19]. Thus, concentrations of 5 mM and 10 mM were used in this study. In 6 out of 9 preparations, the initial effect of doxapram at 5 mM resulted in an increase in the amplitude of the EJP followed by a decrease in the amplitude. A representative response in the amplitude of the 25th EJP in a train of 25 pulses at 40 Hz is shown over time with exposure to doxapram (5 mM) (Figure 1).

**Figure 2:**
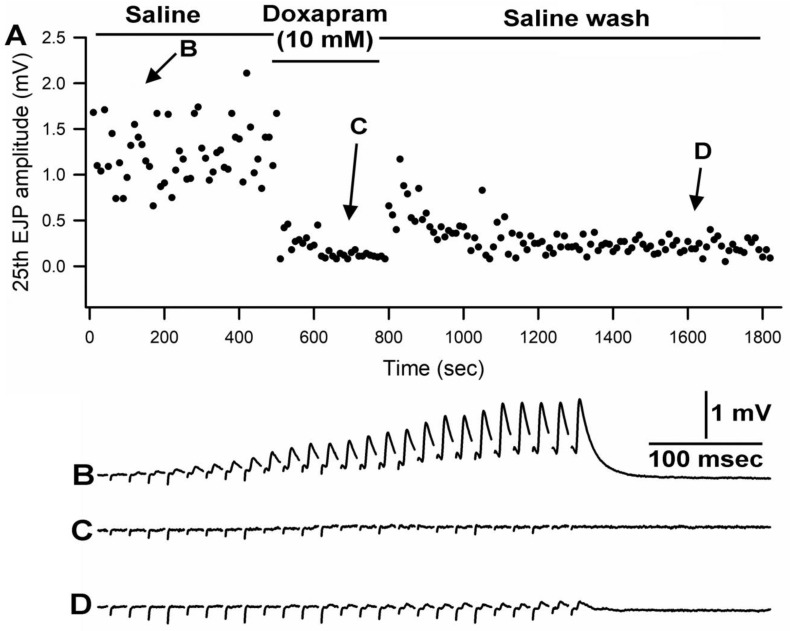
Doxapram at 10 mM rapidly depresses synaptic transmission followed by no recovery with rinsing in saline. (**A**) The amplitude of the 25th EJP in a 40Hz stimulus train over time before, during, and after exposure to doxapram. (**B**–**D**) are representative responses obtained at the times indicated in (**A**).

Doxapram at 10 mM rapidly depressed the EJP amplitudes throughout the entire stimulus train. Even with short exposures of 1 to 2 min, the responses did not recover after flushing the bath with fresh saline multiple times. A representative response in the amplitude of the 25th EJP in a train of 25 pulses at 40 Hz is shown over time with exposure to doxapram (10 mM) (Figure 2).

**Figure 3:**
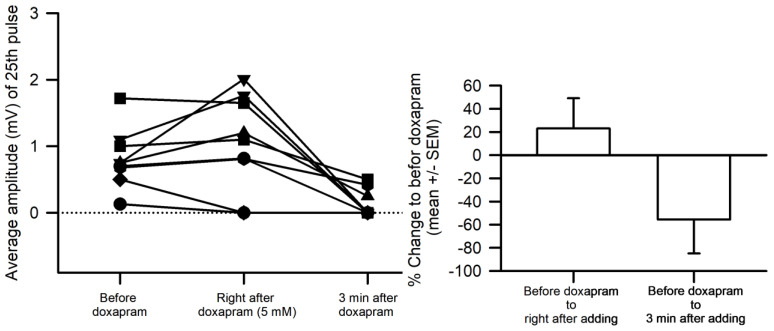
Doxapram, within 30 s of exposure at 5 mM, transiently increased the EJP amplitude of the 25th response in 6 of 9 preparations. Within 30 s and with continual exposure, doxapram decreased the amplitude further than the initial responses prior to exposure. After 3 min, the amplitudes had significantly decreased (Sign test was used, *p* < 0.05). Each line with symbols was an individual preparation.

Since the effects of doxapram at 10 mM were so pronounced, it was decided to use 5 mM for future investigations when examining the ability of doxapram to block any action by LPS. In only 2 out of 9 preparations, doxapram at 5 mM depressed the amplitude of the EJPs quickly and the amplitudes of the 7 of the 9 preparations were still pronounced (Figure 3). The overall percent change in the amplitudes was not significant after immediately applying doxapram due the wide variation in responses (Normality Test Shapiro–Wilk passed, Paired *t*-test, *p* > 0.05, t = −1.765 with 9 degrees of freedom.) However, after 3 min of exposure, the amplitudes significant decreased (due to some values at zero, the data were not normally distributed, so a Sign test was used, *p* < 0.05). Thus, doxapram depressed synaptic transmission.

### 3.2. The LPS Can Dampen the Effect of Doxapram

**Figure 4:**
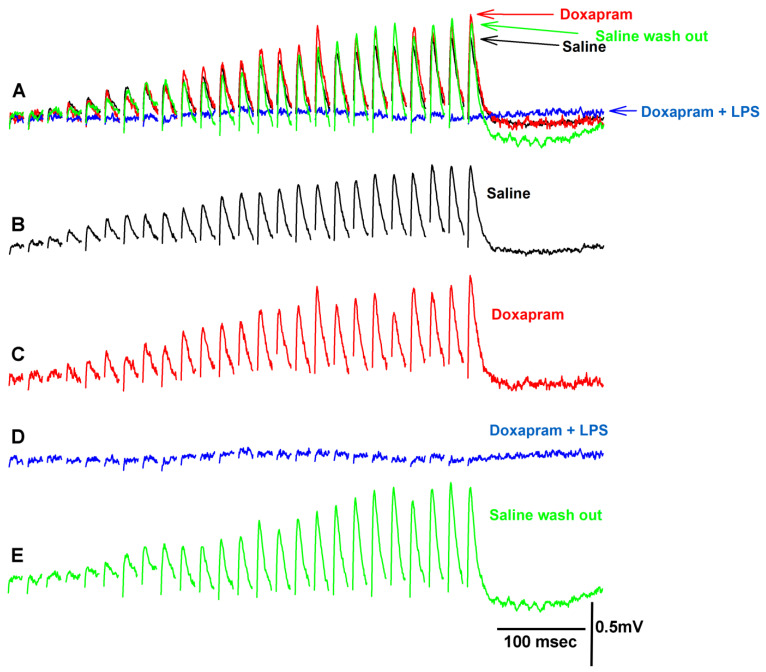
Representative traces of the exposure to doxapram and a combination of doxapram and LPS on synaptic transmission. (**A**) is superimposed traces of the EJPs for each condition highlighted below. (**B**) Saline alone. (**C**) Upon exposure to doxapram. In this example, there was an immediate increase in amplitude of the EJPs after switching the bath. The enhancement in the EJP amplitude after three minutes was depressed as shown in (**D**). (**D**) The cocktail of doxapram (5 mM) and LPS (500 µg/mL) depressed the EJPs. (**E**) After rinsing the preparation three times with saline, a recovery of the EJP amplitudes was possible.

Prior investigations illustrated that LPS from various strains of bacteria enhances the amplitude of the EJP at the crayfish NMJ [11,16,17,28]. At the larval *Drosophila* NMJ, doxapram partially blocked the effect of LPS on increasing the heart rate in larval *Drosophila* [29]. Doxapram (10 mM) alone depressed the heart rate in larval *Drosophila*, depolarized the larval body wall muscles, and caused spontaneous firing of the motor neuron producing random EJPs [19]. However, doxapram at 5 mM or 10 mM did not depolarize the crayfish opener muscle or result in spontaneous EJPs as shown in the investigations herein. In fact, it appears that evoked synaptic transmission of the motor neuron was initially enhanced by a larger EJP amplitude in the majority of preparations followed by depression with exposure to doxapram (5 mM). Even if the preparation showed an initial enhancement of synaptic transmission by doxapram, within the next three minutes, evoked synaptic responses would be depressed and in some cases, completely blocked. Figure 4 depicts a preparation in which the synaptic responses were first enhanced (Figure 4C) and then fully blocked in response to LPS presented as a cocktail with doxapram (Figure 4D). This particular preparation was able to regain its ability of evoked responses after flushing the preparation with saline to remove the doxapram and LPS (Figure 4E).

**Figure 5:**
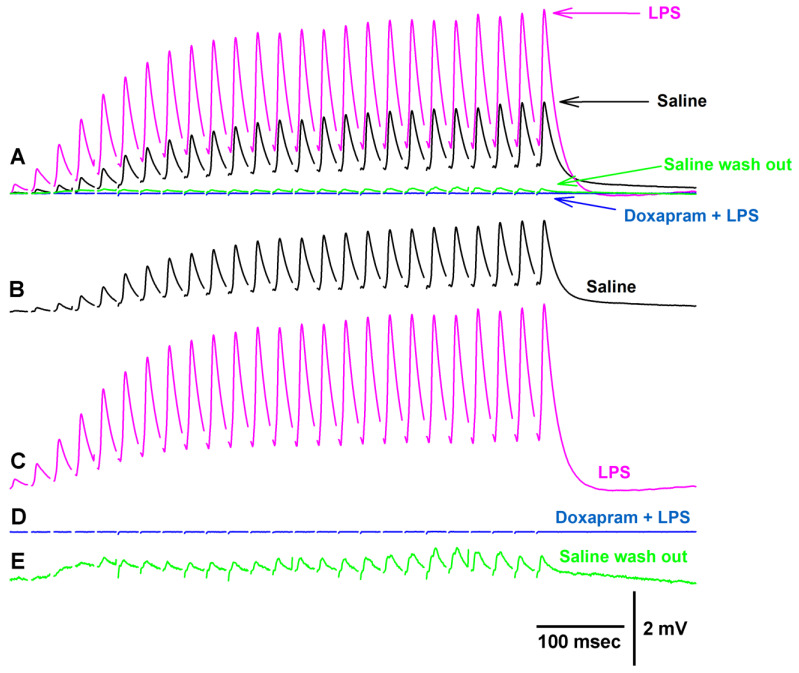
Representative traces of the exposure to LPS and a combination of LPS and doxapram on synaptic transmission. (**A**) is superimposed traces of the EJPs for each condition highlighted below. (**B**) Saline alone. (**C**) Upon exposure to LPS. In this example, an increase was immediate after switching the bath to one containing LPS. (**D**) The cocktail of LPS (500 µg/mL) and doxapram (5 mM) depressed the EJP amplitudes. (**E**) After rinsing the preparation three times with saline, a slight recovery of the EJP amplitudes was possible.

Altering the exposure of the preparation to first LPS and then doxapram (Figure 5) in the majority of cases resulted in an enhancement in the amplitude of the EJP (Figure 5C), followed by depression with the cocktail of LPS and doxapram (Figure 5D). This particular preparation sightly recovered after flushing the bath with fresh saline (Figure 5E).

**Figure 6:**
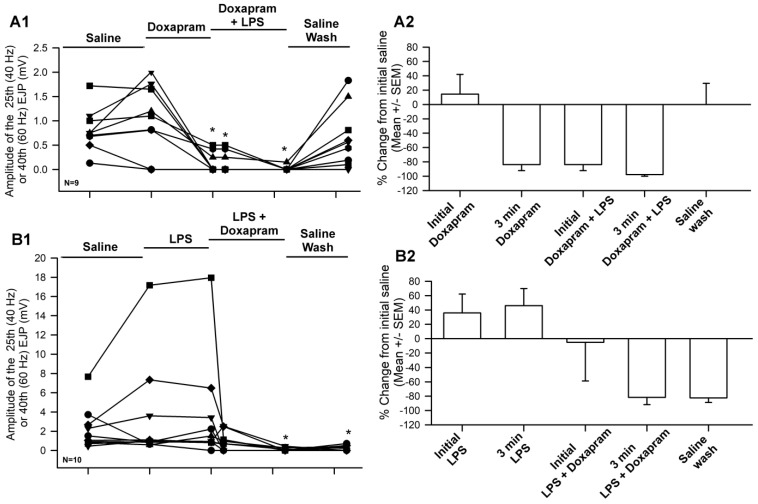
The amplitude of the EJPs from either 40 Hz (25 stimuli) or 60 Hz (40 stimuli) stimulus trains for two different paradigms in exposure to LPS and doxapram. Saline for 3 min, doxapram or LPS for 3 min, doxapram + LPS for another 3 min, and then 3 min of saline wash. (**A1**) The responses when doxapram was initially exposed to the preparation prior to the cocktail of doxapram and LPS and followed by a wash out of the bath with saline. (**A2**) The percent change from the initial saline exposure for each preparation shown in A1 (mean +/− SEM). (**B1**) The responses when LPS was initially exposed to the preparation prior to the cocktail of LPS and doxapram and followed by a wash out of the bath with saline. (**B2**) The percent change from the initial saline exposure for each preparation shown in B1 (mean +/− SEM). LPS (500 µg/mL) and doxapram (5 mM). Each line represents individual preparations. The asterix * in (**A1**,**B1**) represent significant differences (Sign rank sum analysis was used to compare significant differences to the initial values in saline, *p* < 0.05).

The overall effect of the cocktail of LPS and doxapram depressed evoked synaptic transmission even after an enhancement by LPS alone (Figure 6). This indicates that doxapram can block the effect of LPS. However, doxapram suppressed evoked synaptic transmission on its own after three minutes Figure 6(A1,A2) and did not allow LPS to enhance evoked synaptic transmission Figure 6(B1,B2). In all cases, doxapram depressed evoked synaptic transmission within three minutes regardless of if LPS was initially exposed to the preparation (N = 9) or if doxapram was initially exposed (N = 9). The data are not normally distributed when the values approach zero; thus, Sign rank sum analysis was used to compare significant differences to the initial values in saline. The asterix * in Figure 6(A1,B1) represents significant differences (*p* < 0.05).

**Figure 7:**
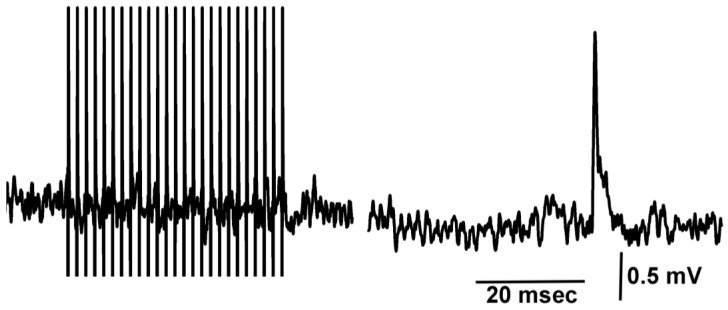
Spontaneous quantal events occur while evoked synaptic responses are blocked by doxapram. A train of 25 pulses delivered at 40 Hz in the presence of doxapram (5 mM) illustrates the blocked evoked responses, while in the same recording trace, a spontaneous quantal event occurred.

Even after evoked synaptic responses were blocked during the exposure to doxapram (5 mM), spontaneous quantal events were observed (Figure 7). Thus, doxapram is not blocking the postsynaptic glutamate receptors.

## 4. Discussion

In this investigation, it was demonstrated that doxapram at 10 mM rapidly depressed evoked synaptic transmission at the glutamatergic synapses of the crayfish NMJ. At 5 mM, doxapram enhanced synaptic transmission briefly and, in some preparations, depressed transmission. In all preparations after three minutes, evoked synaptic depression was severely depressed, as shown by a reduced amplitude in the evoked responses. Additionally, doxapram (5 mM) depressed the excitatory impact of LPS from *Serratia marcescens*. When incubated in doxapram, prior to exposure of the cocktail of LPS and doxapram, the response was blunted and the increased amplitude of the EJPs normally observed with LPS did not occur.

The concentration of 5 mM is likely a transitional concentration where some preparations are susceptible to depression while the majority of others are enhanced followed by depression. Interestingly, the enhanced responses would gradually be depressed showing a reduced facilitation in the amplitude of the EJPs within the train of responses. Given that spontaneous quantal events can still occur while the evoked responses are blocked, it is suggested that doxapram is having an effect at the presynaptic side by potentially blocking Ca^2+^ entry. This could occur either by directly blocking voltage gated Ca^2+^ channels, or by preventing an action potential from occurring or reaching the nerve terminal, or possibly a combination of effects. Intracellular recording in the axons of the motor neurons would help to differentiate the mechanisms of action of doxapram.

If doxapram blocks ion channels in the axon or presynaptic terminal, it is not surprising that the enhanced response by LPS would be dampened. However, the mechanism by which LPS enhances synaptic transmission was not elucidated in the initial report by Parnas et al. [28] or in later reports [11,16,19]. Thus, if LPS from *Serratia marcescens* is promoting Ca^2+^ through presynaptic voltage gated ion channels, then it is possible that LPS and doxapram might be targeting the same site of action. Imaging with Ca^2+^ indicators within the axon terminal or intracellular recording of the action potential during exposure to LPS or doxapram would help to delineate the mechanism of action. One would assume that if LPS causes the voltage gated Ca^2+^ channels to open or remain open for a longer period while inducing action potentials in the axon, there would be an increase in the occurrences of spontaneous quantal events. However, this was not observed in an earlier study using the same preparation [11,16]. One mechanistic possibility is that LPS may reduce the inactivation of voltage-gated Na^+^ channels in the axons, which would broaden the action potential and prolong the depolarization of the terminal during evoked conditions002C allowing more Ca^2+^ influx during short periods, without promoting a rise in the frequency of spontaneously occurring vesicles.

Doxapram (5 and 10 mM) at the larval *Drosophila* NMJ rapidly depolarizes the muscle, maintains the muscle in a depolarized state, and causes the motor nerve to fire and produce random evoked EJPs [19]. Thus, doxapram depolarizes both the muscle and the motor nerve terminal at the larval *Drosophila* NMJ. The action of doxapram at the larval *Drosophila* NMJ concurs with the mechanism of action for doxapram as an antagonist to K2P channels. The K2P channels are responsible for maintaining the resting membrane potential of cells, as they serve as the K^+^ leak channels in membranes to maintain the potential close to the equilibrium potential for K^+^ ions [2,18]. It has been reported that doxapram inhibits TASK, a K2P subtype in acid-sensitive mammals [30,31]. An acidic saline 7.1 reduced to 6.5 and even 6.0 will depolarize the larval *Drosophila* muscle and crayfish muscle [11,32]. Since the skeletal muscle of larval *Drosophila* depolarizes in acidic conditions and upon exposure to doxapram [11,19], it is likely that larval muscle expresses a TASK-like K2P channel. However, since crayfish muscle depolarizes in acidic conditions and a lowered pH depresses synaptic transmission, there may be acid sensitive K2P channels present in the membrane of crayfish muscle [32]. The depression of synaptic transmission seen in acidic conditions may be more of an effect on the motor nerve terminal. If the terminal is depolarized and inactivates voltage-gated Na+ channels, then there would be fewer to open during an evoked action potential and likely a smaller amplitude and narrower action potential. Doxapram does not strongly influence the resting membrane potential of crayfish muscle. It is possible the K2P channel subtype is different in crayfish muscle than that of larval *Drosophila* muscle or carotid bodies of mammals. In fact, genomic studies of K2P channel subtypes have yet to report on the subtypes present in crustaceans [2,18,33,34].

LPS at the larval *Drosophila* NMJ may acutely stimulate the K2P channels on the muscle since the muscle rapidly hyperpolarizes in the presence of LPS and this hyperpolarization is rapidly blocked by doxapram [11]. The LPS from Gram-negative bacteria mediates the cellular response through the PGRP-LE/PGRP-LC receptors and the IMD cascade through the NF-κB factor Relish with the genomic response to produce AMPs in insects [5,16,35,36]. However, these cellular pathways have yet to be investigated in crustaceans. There is a caveat when using commercially available LPS from whole bacteria or lysed bacteria, as other cellular components may be present, such as repeats-in-toxin (RTX), which can make pores in the membrane of cells, as well as lipoproteins, glutamate, and even adenosine [37,38,39,40,41,42]. Thus, the effects assumed to be due to LPS may be due to a combination of cellular constituents. It is difficult to obtain ultra-purified LPS for the various bacterial stains as only a few are commercially available and there does not appear an ultrapure LPS form for *Serratia marcescens.* There is and ultrapure form for *Salmonella enterica* serotype Minnesota, which has yet to be examined at the *Drosophila* or crayfish NMJs for physiological effects. If the animals are exposed to bacteria and it becomes blood (hemolymph) borne, then all the constituents of the bacterial membrane and cytosolic matter would be exposed to the tissues. Thus, even if not a pure form of LPS is examined, the responses do provide some insight into the potential physiological reactions in a natural setting. Given the crayfish used in this study are caught from the wild and do possess some cuticle damage in spots as well as likely having a potential mix of bacterial strains in the hemolymph or LPS leaking from the gastrointestinal tract into the hemolymph, then responses to the isolated NMJs of LPS could be altered due to being previously exposed to LPS. These wild caught crayfish are also noted to carry internally parasitic cysts which may alter immune reactions. The use of wild caught animals does make it difficult to control for some variables which could impact investigations related to immune or direct LPS exposures.

There are still many unanswered questions as to the direct action of doxapram and LPS by themselves on the crustacean preparations, as well as in insect preparations including *Drosophila*. However, as more information is gained, the better our understanding will be in characterizing the response to bacterial infections and cellular responses in various organisms as well as how pharmacological agents can impact the responses. Perhaps the NMJs of the crayfish can aid in serving as a model for this purpose as well as developing a better understanding in the actions of doxapram since it has been and is still used clinically for humans and in veterinary medicine [43,44,45,46,47].

## 5. Conclusions

In summary, the compound doxapram at 10 mM rapidly depressed evoked synaptic transmission while 5 mM was slightly mixed, with some first enhancing transmission and depressing over time at these glutamatergic synapses. Doxapram did retard the excitation of evoked transmission normally observed with exposure to LPS from *Serratia marcescens*. Although, the concentrations used of doxapram are high in this investigation, the proof of concept is that the excitation induced by LPS is blocked. Potential structural modifications of doxapram might provide more potent forms. Given doxapram is a blocker of the acid-sensitive K2P channel subtype, it is of interest to determine if this is indeed the mechanism blocking the LPS response as proposed for larval *Drosophila*.

## Data Availability

All data generated or analyzed during this study are included in this published article.
